# A Case of Chorea Treated by Chloroform

**Published:** 1852-05

**Authors:** Wm. A. Milner

**Affiliations:** Union Parish, Louisiana


*A Case of Chorea treated by Chloroform.* By Wm. A. Milner, M. D., of Union Parish, Louisiana.—I believe it is generally admitted that but little more is known of the nature of this disease, than that it is a functional derangement of the nervous system. It is, therefore, not my design to advance pathological views upon a snbject so imperfectly understood by abler and more experienced practitioners, but merely to give the treatment of a single case, which will, perhaps, not be uninteresting.

I am aware, too, that the prognosis of uncomplicated chorea is generally favorable, there being instances of spontaneous recoveries; nevertheless, it is a very ugly, and I may say formidable disease, and one which excites the greatest interest and solicitude on the part of friends and parents.

June 5. My attention was called to the daughter of Mr. L., aged nine years. She was able to run about, but there was irregular motion of the hands, and some slight twitching of the muscles of the lips and face; she was yet able to feed herself—complained of some headache. She was taken from school two months previous to my seeing her, from complaing of headache and general, but slight indisposition. *Prescription*—Active purge of rhei, colocynth and calomel every third night. Being rather anæmic, put her on Valet's ferruginous pill, three times per day.

June 8. Bowels are well acted upon; but the case has grown rapidly worse. It seems that every voluntary muscle in the whole system is in motion; deglutition is performed with great difficulty—requires two persons to hold her up. Average sleep in twenty-four, one to two hours, in very short naps. *Prescription*—$\frac{1}{4}$ gr. sulph. morph., repeat every hour until sleep is induced; applied blister, two and a half by six inches, over dorsal region; sulph. zinc, one grain three times per day.

June 10. My patient has eaten nothing of consequence for two days; stomach very irritable; the zinc was rejected—repeated, and rejected, for two days; morphine seems to aggravate, even in one grain doses, instead of quieting and inducing sleep. Blister drew well, and is considerably irritated from the incessant motion, which makes me regret having put it on. *Prescription*—Oil and turpentine to move the bowels; stop the zinc, and gave comp. tinct. gentian ʒj., and ℥j. infusion snake root, three times per day, with 1 gr. ext. hyoscy. at night, and repeat once, in an hour, if required.

June 11. Patient rested better last night, stomach is quieted, and desires to eat Continued same treatment.

June 12. Patient passed a restless night; has some erysipelatous spots on hands and feet, which I attribute to hyoscy., having taken 2 grs. last night. The stomach is quiet; eats soup or gruel with difficulty; takes nothing solid. The muscular disturbance is still very great; no appreciable amendment.

Dr. Milner then resorted to chloroform, and by July 5th his patient was quite clear of all symptoms of chorea. The cure he attributes to the inhalation of chloroform.

[*Southern Medical and Surgical Journal.*

